# Clinical outcome and cost of intravenous, OPAT, and oral treatment of Gram-negative bacteremia

**DOI:** 10.5339/qmj.2025.108

**Published:** 2025-12-10

**Authors:** Mas Chaponda, Farah Maher, Joanne Daghfal, Adila Shaukat, Walid Al-Wali, Manaf Kadhim Hashim Al-Obaidi, Muna Al Maslamani

**Affiliations:** 1 Hamad Medical Corporation, Doha, Qatar *Email: mchaponda@hamad.qa

**Keywords:** Gram-negative bacteremia, OPAT, antibiotics, inpatients, intravenous therapy, length of stay

## Abstract

**Background:**

Gram-negative bacteremia (GNB) causes substantial morbidity, mortality, prolonged hospitalization, and readmissions worldwide. Standard practice recommends initial intravenous (IV) antibiotics, but many clinically stable patients now complete therapy through outpatient parenteral antimicrobial therapy (OPAT), which may improve antimicrobial stewardship and reduce costs. This study aims to compare clinical and economic outcomes of inpatient versus OPAT-managed GNB.

**Methods:**

We conducted a retrospective observational cohort study of all adults with GNB treated at a 320-bed general hospital from July 2021 to July 2022. We excluded patients younger than 18 years, blood culture contaminants, and patients with Gram-positive or mixed infections. Patients were assigned to four final treatment pathways: inpatient IV only, hospital-based OPAT IV room (OPAT-IV), home-based IV therapy (Home-IV), or early oral switch. Primary outcomes were 30-day mortality and bacteremia recurrence; secondary outcomes were length of stay and treatment cost.

**Results:**

We identified 125 patients (mean age, 56 years; 51.2% female). Most isolates originated from urinary (54.4%) or intra-abdominal (23%) sources. Pan-sensitive organisms accounted for 57.6% of cases; extended-spectrum β-lactamase producers, 35.2%; and multidrug-resistant organisms, 7.2%. Thirty-seven patients (29.6%) completed IV therapy as inpatients, 16 (12.8%) in OPAT-IV, 13 (10.4%) in Home-IV, and 59 (47%) were discharged on oral antibiotics. Nine deaths (24.3%) occurred in the inpatient group and one (7.7%) in Home-IV; no deaths occurred in OPAT-IV or oral-therapy groups. Readmissions within 90 days were uncommon (≤7.7% across groups). Mean antibiotic durations were 13.0 days (inpatient), 11.3 days (OPAT-IV), 10.6 days (Home-IV), and 7.7 days (oral). OPAT pathways shortened hospital stays and reduced drug and bed-day costs.

**Conclusion:**

OPAT provided safe, effective treatment for selected patients with GNB, lowering mortality, shortening length of stay, and reducing costs compared with continued inpatient care. Careful patient selection and early IV-to-oral transition can maximize these benefits while maintaining favorable clinical outcomes.

## 1. INTRODUCTION

Gram-negative bacteremia (GNB) is the most common life-threatening infectious condition associated with significant morbidity, mortality, and adverse patient experiences. The standard treatment has historically involved intravenous (IV) antibiotic therapy for 10 to 14 days.^1^ However, treatment durations have gradually shortened,^2^ with increasing consideration for early transition to oral antibiotics, often followed by extended oral regimens.^3^ The severity and type of infection typically guide the route of antimicrobial administration. Many clinicians continue to prefer IV antibiotics for serious infections.

Outpatient parenteral antimicrobial therapy (OPAT) is a treatment option for clinically stable patients who still require parenteral therapy.^4^ OPAT has become more feasible with the availability of antimicrobials suitable for once- or twice-daily dosing.^4^ Although OPAT has demonstrated safety in various Gram-positive infections,^1^ few studies have specifically evaluated its use in GNB.

OPAT was first introduced in the United States in the mid-1970s as a cost-containment strategy for inpatient IV antibiotic therapy.^5^ By reducing inpatient length of stay, OPAT has helped decrease healthcare-associated complications, hospital costs, and patient burden.^6–8^

As OPAT becomes more widely implemented, it is increasingly important to define which patients and clinical scenarios are appropriate for this modality. There is also an opportunity to enhance antimicrobial stewardship and to develop quality indicators—structural, process, and outcome measures—to ensure safe care delivery and evaluate OPAT’s impact.^9^ While OPAT is commonly used to complete antibiotic courses after initial hospitalization, it also carries potential risks, including reduced clinical oversight and complications related to IV access.^10^

The primary aim of this study is to compare clinical outcomes in patients with GNB treated with either continued inpatient hospitalization, OPAT following initial hospitalization, or early transition to oral antibiotics. Secondary outcomes are the length of stay, duration of antibiotic therapy, and total cost of antibiotic treatment and hospitalization. We expect clinical outcomes to be good in OPAT patients whilst reducing costs and maintaining patient safety.

## 2. METHODS

This descriptive, retrospective, observational cohort study included adult patients (aged ≥18 years) diagnosed with GNB between July 2021 and July 2022 at a general hospital with more than 320 beds. This period represents the first year of the hospital’s OPAT program. Patients were eligible for OPAT if they were clinically stable (e.g., afebrile and with stable vital signs) and did not require ongoing inpatient care. The hospital serves a diverse patient population, including transit airline passengers.

Antibiotics were administered through two OPAT modalities: (1) a home-based mobile team, primarily for older adults or patients with limited mobility, and (2) an on-site IV room staffed by hospital nurses, pharmacists, a program coordinator, and physicians, including infectious disease consultants. The program enrolls approximately 10 new patients per week, with 90% referred after hospitalization from Internal Medicine, General Surgery, or Urology, and 10% referred directly from the Emergency Department. The OPAT service does not accept pediatric patients (i.e., those aged <18 years). The hospital’s OPAT protocol is designed for clinically stable patients (i.e., those with stable vital signs) who no longer require inpatient therapies, such as physiotherapy, and who are otherwise eligible for discharge but require completion of intravenous antibiotic therapy.

Eligible patients were stratified into four groups based on their final antibiotic treatment pathway: inpatient IV therapy only, OPAT IV therapy, home-based IV therapy, or early oral switch. Exclusion criteria included age <18 years, blood culture isolates deemed contaminants by both a clinician and microbiologist, and infections involving Gram-positive or polymicrobial organisms.

Data collected included demographic characteristics, comorbidities, infection source, resistance patterns, antibiotic type, and treatment duration. The primary outcome was 30-day all-cause mortality. In cases of gram-negative bacteraemia, repeat blood cultures are generally not recommended as a routine practice due to their low yield. So, the secondary outcomes were 90-day readmission related to the initial infection and recurrence of bacteremia with the same organism within 30 days. Costs were assessed using Wholesale Acquisition Cost (WAC) for antibiotics and estimated hospital bed-hour expenses.

A binary logistic regression model assessed predictors of 30-day mortality. The primary independent variable was treatment setting (outpatient vs. inpatient), with age and diabetes included as covariates. The dependent variable was 30-day mortality (yes/no). Outpatient care was defined as the completion of therapy via OPAT IV room, home IV therapy, or early oral switch.

Results were expressed as odds ratios (ORs) with 95% confidence intervals (CIs). All analyses were performed using Stata Statistical Software: Release 16.1. (College Station, TX: StataCorp LLC.).

Data were extracted from the electronic patient record by a multidisciplinary research team comprising internal medicine physicians, infectious disease specialists, medical microbiologists, a clinical pharmacist, and an epidemiologist. Coded data were entered into Microsoft Excel and exported to STATA for analysis. Categorical data are reported as frequencies and percentages; continuous data are presented as means with standard deviations. The Fisher’s exact test was used for categorical variables, and the Mann–Whitney U test was used for continuous variables.

## 3. RESULTS

A total of 125 patients were diagnosed with GNB during the one-year study. The mean patient age was 56 years. Most patients were admitted to medical wards (72.8%), followed by surgical and urology wards (16%), obstetric wards (4.8%), the medical intensive care unit (2.4%), the surgical intensive care unit (1.6%), the emergency department (1.6%), and the burns unit (0.8%).

### 3.1 Demographics and comorbidities

Of the 125 patients, 64 (51.2%) were women and 61 (48.8%) were men. The cohort included eight pregnant women (12.5%), 59 patients with diabetes (47.2%), seven patients receiving dialysis for end-stage renal disease (5.6%), and 13 patients with chronic kidney disease (10.4%).

### 3.2 Source of infection

The most common source of bacteremia was urinary tract infection, identified in 68 patients (54.4%), followed by intra-abdominal infections in 29 patients (23.2%). Source control (e.g., stenting or drainage) was achieved in 4.6% of these cases. Other sources included respiratory (10.4%), soft tissue (4.8%), and unknown origins (7.2%; Table 1).

### 3.3 Antibiotic resistance

A total of 72 patients (57.6%) had infections with antibiotic-sensitive organisms. Extended-spectrum beta-lactamase (ESBL)-producing organisms were identified in 44 patients (35.2%), while multidrug-resistant organisms (MDROs) were found in nine patients (7.2%). Among the ESBL cases, 20 patients received ertapenem through the OPAT service. The remaining ESBL and all MDRO cases were managed as inpatients.

### 3.4 Disposition

Among the 125 patients, 37 (29.6%) completed IV antibiotics as inpatients. Fifty-nine patients (47.0%) were discharged on oral antibiotics after an average inpatient stay of six days. The OPAT IV room managed 16 patients (12.8%), and 13 patients (10.4%) were treated with home IV antibiotics administered by a mobile team.

### 3.5 Clinical outcomes

Inpatient care was associated with the highest mortality, with nine deaths (24.3%). One death occurred in the home IV group (7.7%), while no deaths were recorded in the OPAT IV room or oral therapy groups (Table 2).

Readmission rates due to recurrence with the same pathogen within 90 days were low overall. The highest rate was observed in the home IV group (7.7%), followed by the inpatient group (5.4%).

### 3.6 Predictors of mortality

Multivariable logistic regression was performed on 116 patients. Outpatient treatment (OPAT IV room, home IV, or oral antibiotics) was independently associated with significantly lower 30-day mortality compared to inpatient treatment (OR, 0.033 [95% CI, 0.004–0.275]; *P* = 0.002). Neither age (OR, 0.978 [95% CI, 0.934–1.025]; *P* = 0.357) nor diabetes (OR, 2.344 [95% CI, 0.409–13.425]; *P* = 0.339) was significantly associated with mortality (Table 3).

### 3.7 Antibiotic duration and modification

The mean total duration of antibiotic therapy was highest in the inpatient group (13.1 days) and lowest in the home oral group (7.7 days). The number of modifications in antibiotic therapy reflected adjustments from empirical treatment to targeted therapy based on culture results and, in outpatient settings, the need for once- or twice-daily dosing (Table 4).

### 3.8 Antibiotic changes

Antibiotic regimens included broad-spectrum agents such as meropenem, ertapenem, piperacillin/tazobactam, ceftriaxone, and cefepime. Narrow-spectrum agents included ampicillin-sulbactam, cefazolin, and amoxicillin-clavulanate.

### 3.9 Cost of medication

Patients managed entirely in the inpatient setting had the highest IV antibiotic costs (mean 998.92 Qatari Riyals), followed by the home IV group (700.90 Qatari Riyals). The home oral group had the lowest medication cost (Table 5).

### 3.10 Cost of management of Gram-negative infections

Table 6 presents the mean costs associated with treatment modalities. Inpatient care incurred the highest average daily cost (2008.81 Qatari Riyals), followed by OPAT IV room visits and home oral therapy.

## 4. DISCUSSION

The efficacy and safety of OPAT for treating Gram-positive infections, including methicillin-susceptible *Staphylococcus aureus*, are well established.^11^ This study contributes to the growing body of evidence supporting the safe use of OPAT in patients with GNB. The primary outcome of 30-day mortality demonstrated a clear benefit for OPAT compared with inpatient treatment. Although this result is not unexpected, given that patients who remained hospitalized were generally sicker and had more comorbidities, it is reassuring that early discharge via OPAT did not result in higher mortality or readmission rates. In the OPAT cohort, most infections originated from the urinary tract or intra-abdominal sources. Adjusted analysis revealed that patients in the outpatient group had approximately 97% lower odds of 30-day mortality compared with inpatients, a statistically and clinically significant finding.

Adverse events associated with OPAT typically fall into two categories: catheter-related and antimicrobial-related. Potential complications of indwelling IV catheters include bloodstream infections, thrombosis, mechanical obstruction, and chemical phlebitis.^12,13^ In this study, no catheter-related complications were reported. This is likely due to the safety protocols in place, such as initiating OPAT only after inpatient evaluation to ensure tolerability. Previous studies have similarly reported low rates of such complications.^14,15^

The rate of hospital readmission due to OPAT-related complications is approximately 9% in some academic settings.^16^ In our study, readmission rates were low across all groups during the first year of OPAT implementation. Establishing a longitudinal registry to monitor OPAT patients would support antimicrobial stewardship and ensure ongoing quality assurance in line with good clinical governance practices.

OPAT offers substantial advantages: it conserves inpatient bed capacity, reduces the risk of healthcare-associated infections, and lowers costs. In addition, it enhances the patient experience by allowing recovery in the comfort of home and promoting early independence.^17^ From a system-wide perspective, OPAT allows hospitals to allocate more resources to patients requiring intensive care.

Cost savings associated with OPAT were significant for individual patients and healthcare systems. Antibiotic costs were lowest in the home oral group, and OPAT treatment was less expensive than inpatient IV therapy. However, it is important to note that certain OPAT regimens can increase pharmacy expenditures. For instance, switching from inpatient vancomycin to daptomycin for Gram-positive infections—often chosen for once-daily dosing convenience—can significantly raise costs.^18^ These findings underscore the need to evaluate whether newer, more expensive agents are being selected appropriately, particularly since a core goal of OPAT is to reduce healthcare expenditures.^19^

Each OPAT delivery model has its strengths and limitations. The hospital-based IV room offers supervised administration with immediate access to medical staff and equipment, but requires dedicated infrastructure and patient travel. Home-based IV care, typically offered to elderly or immobile patients, enhances convenience but was not costed in this study as it falls under primary care services. Although home administration is preferred by many patients,^20^ this model may not be scalable for younger, mobile patients unless self-administration training is implemented.

Despite its benefits, OPAT presents challenges. It can be labor-intensive for healthcare teams and may involve IV access, frequent laboratory monitoring, and patient travel complications. A common concern is the overuse of broad-spectrum antibiotics in OPAT settings, often selected for convenience despite narrower-spectrum alternatives requiring multiple daily doses.^21^ This practice risks fostering antimicrobial resistance, including multidrug-resistant organisms. Expanding stewardship programs to include OPAT is essential.^22^ Evidence suggests that certain broad-spectrum OPAT regimens could be substituted with narrower-spectrum agents without compromising efficacy.^23^

Oral antibiotic therapy offers key advantages over IV treatment, including avoidance of catheter-related complications, lower drug costs, and eliminating associated hidden costs (e.g., professional administration and equipment). Transitioning patients to oral antibiotics can facilitate earlier discharge and reduce healthcare resource utilization.^24^

Emerging evidence supports shorter durations of IV therapy and earlier IV-to-oral switch therapy (IVOST) for many infections, including respiratory, intra-abdominal,^25^ and genitourinary infections.^1^ In some cases, short-course therapy may eliminate the need for OPAT. IVOST, when appropriately implemented, is safe and convenient. Patient selection should be based on clinical response, immune status, comorbidities, gastrointestinal function, and ability to adhere to oral therapy.^26^ Knowledge of local resistance patterns and microbiological data is also essential. Notably, most patients in our cohort were discharged on oral antibiotics.

However, studies from the Middle East and North Africa region suggest that oral antibiotic therapy is often unnecessarily prolonged following discharge.^3^ This approach may increase the risk of adverse outcomes such as Clostridioides difficile infection and the emergence of antimicrobial resistance.

### 4.1 Limitations

This study has several limitations. First, it is a retrospective observational study with a relatively small sample size. Given this was an observational descriptive study, we did not perform a formal sample-size calculation. Furthermore, all subgroup analyses were underpowered to draw associations, and our findings will need to be further confirmed in a larger prospective study. Also, the lack of baseline severity markers potentially biases our mortality data. However, we expect that carefully chosen stable patients are suitable for OPAT and will have better mortality outcomes than those who remain unwell in the hospital. The absence of documented clinical reasoning for selecting broad-spectrum antibiotics when narrower-spectrum options were available limited our ability to assess prescribing decisions. As OPAT use expands and more standardized pathways are implemented, future studies should evaluate the role of antimicrobial stewardship in this setting and assess potential cost-saving opportunities.

Due to the limited sample size, this study could not confirm any statistically significant differences in collateral outcomes, such as the emergence of *Clostridioides difficile* infection, which has been reported in other studies following OPAT use.^27^ Additionally, a longer follow-up period would be valuable, as one-year mortality may be a more meaningful clinical endpoint than 30-day mortality, as suggested by previous cohort studies.^10^

For the economic analysis, drug cost estimates were based on WAC due to contractual confidentiality. Although this approach does not capture true acquisition costs, the overall conclusions regarding the cost-effectiveness of OPAT are unlikely to change if actual purchase prices were used. The analysis also did not account for the costs associated with monitoring therapy, managing adverse events, or indirect operational costs such as hospital bed usage, home-nursing, laboratory monitoring, and transport to and from the hospital. It is possible that these additional costs would compare with hospitalization costs, but this would have to be confirmed in a comprehensive cost analysis model that includes all hidden costs.

The costs associated with the home mobile team were excluded, as they are managed through primary care services. Furthermore, the study could not calculate bed days saved due to the lack of ward-specific monthly occupancy data. Accurate calculation of bed days requires the number of occupied adult acute care beds and the total number of available beds each month.

This study was conducted at a single center, which may limit the generalizability of the findings. A prospective study would be valuable to capture clinician-reported reasons for regimen selection, patient-reported outcomes, and satisfaction metrics.^28^ We recommend that future efforts focus on achieving an optimal balance between short-course OPAT and oral therapy, where appropriate, in alignment with global trends favoring oral antimicrobial treatment.^25^

Antimicrobial stewardship in the OPAT setting could be improved by prioritizing less frequently dosed agents, such as long-acting glycopeptides; restricting and tailoring susceptibility testing based on OPAT requirements; and incorporating routine laboratory monitoring and safety oversight. Rapid molecular diagnostics will likely be increasingly important in optimizing antimicrobial selection and minimizing unnecessary treatment.

Robust clinical governance is essential to ensure the safety and efficacy of OPAT programs. Institutions should adopt evidence-based clinical protocols, integrate OPAT into standardized care pathways, and implement routine clinical audits supported by dashboards that track case volume, patient mix, treatment outcomes, adverse events, and indicators of collateral damage. These efforts should be multidisciplinary and focused on continuous quality improvement.

## 5. CONCLUSION

This study demonstrates that OPAT is a clinically effective, practical, and cost-efficient approach for managing selected patients with GNB. Careful patient selection, guided by source control and clinical stability, is critical to successful OPAT outcomes. Early transition to oral antibiotics or outpatient IV therapy may provide an alternative to prolonged hospitalization, offering favorable clinical results and reduced healthcare costs. Adopting diagnostic stewardship, including optimized use of microbiology laboratories and rapid molecular testing, can support appropriate antimicrobial selection and reduce the duration of inappropriate therapy. These strategies ultimately contribute to improved patient outcomes and more sustainable healthcare delivery.

## LIST OF ABBREVIATIONS

**Table:** 

CI	Confidence interval
ESBL	Extended-spectrum beta-lactamase
GNB	Gram-negative bacteremia
IV	intravenous
IVOST	IV-to-oral switch therapy
MDRO	Multidrug-resistant organisms
OPAT	Outpatient antimicrobial therapy
OR	Odds ratio
WAC	Wholesale acquisition cost

## ETHICAL APPROVAL

The study was approved under expedited review by the Investigational Review Board of Hamad Medical Corporation (MRC-01-22-546).

## AUTHORS’ CONTRIBUTIONs

All authors contributed to the conception and design of the study and drafted the manuscript, including giving final approval to the manuscript version submitted for publication. All authors read and approved the final manuscript.

## ACKNOWLEDGMENTS

The authors thank Dr Hala Zaki Abdullah Sonallah, PharmD, Dr Amira Fawaz Bitar, Dr Asmaa Salah Ahmed Abdelkarim, and Dr Fager Gamal Ibrahim Abdelbagi for their assistance with data collection.

## CONFLICT OF INTEREST

The authors declare no conflict of interest.

## Figures and Tables

**Table 1.** Source of bacteremia (N = 125).

Source of bacteremia	*N* (%)
Urine	68 (54.4%)
Abdomen	29 (23.2%)
Chest	13 (10.4%)
Unknown	9 (7.2%)
Soft tissue	6 (4.8%)

**Table 2.** Clinical outcomes by treatment group.

Outcome	Home IV (*N* = 13)	Home oral (*N* = 59)	Inpatient (*N* = 37)	IV room (*N* = 16)	*P* value	Test
Deceased, *n* (%)	0 (0%)	1 (1.69%)	9 (24.32%)	0 (0%)	0.015	
Alive, *n* (%)	12 (92.31%)	54 (91.53%)	26 (70.27%)	16 (100%)	
Readmission, *n* (%)	1 (7.69%)	1 (1.69%)	2 (5.41%)	0 (0%)	
Left country *n* (%)	0 (0%)	1 (1.69%)	0 (0%)	0 (0%)	
Unknown *n* (%)	0 (0%)	2 (3.39%)	0 (0%)	0 (0%)	

IV: Intravenous.

**Table 3.** Logistic regression analysis for 30-day mortality.

Variable	OR	95% CI	*P* value
Outpatient vs. Inpatient	0.033	0.004–0.275	0.002
Age (per year)	0.978	0.934–1.025	0.357
Diabetes	2.344	0.409–13.425	0.339

OR: Odds ratio; CI: Confidence interval.

**Table 4.** Duration of antibiotic therapy (in days).

Treatment duration	Home IV, *N* = 13, *n* (%)	Home oral, *N* = 59, *n* (%)	Inpatient, *N* = 37, *n* (%)	IV room, *N* = 16, *n* (%)	*P* value
Initial duration of antibiotics	3.46 (2.57)	3.97 (3.16)	6.22 (5.86)	3.00 (2.42)	0.016
Secondary duration of antibiotics	2.85 (3.08)	2.95 (3.47)	5.84 (7.40)	3.13 (1.89)	0.031
Tertiary duration of antibiotics	1.08 (2.14)	0.90 (1.86)	2.70 (5.76)	1.50 (3.08)	0.12
Fourth duration of antibiotics	0.00 (0)	0.19 (1.43)	0.11 (0.66)	0.00 (0)	0.89
Fifth duration of antibiotics	0.00 (0)	0.00 (0)	0.51 (2.18)	0.00 (0)	0.19
Duration of IV (inpatient)	6.23 (2.45)	6.10 (3.88)	11.84 (10.32)	5.94 (2.84)	<0.001
Duration of IV (outpatient)	1.46 (2.82)	0.56 (1.85)	0.24 (0.80)	5.44 (2.56)	<0.001
Duration of IV (home mobile)	2.38 (4.96)	0.08 (0.65)	0.00 (0)	0.00 (0)	<0.001
Total duration of treatment in days	10.69 (3.09)	7.71 (4.80)	13.08 (10.09)	11.38 (2.99)	0.002

IV: Intravenous.

**Table 5.** Cost of antibiotics by treatment group (Qatari Riyals).

Treatment cost	Home IV, *N* = 13, *n* (%)	Home oral, *N* = 59, *n* (%)	Inpatient, *N* = 37, *n* (%)	IV room, *N* = 16, *n* (%)	*P* value
Initial cost of IV vials	268.59 (390.66)	175.22 (198.22)	497.47 (624.09)	111.87 (136.41)	<0.001
Secondary cost of IV vials	357.44 (482.11)	131.53 (190.51)	316.60 (495.26)	312.06 (381.65)	0.035
Tertiary cost of IV vials	74.87 (224.18)	70.00 (234.66)	167.85 (395.97)	243.98 (500.44)	0.21
Fourth cost of antibiotics	0.00 (0)	0.18 (1.41)	7.06 (42.91)	0.00 (0)	0.52
The fifth cost of antibiotics	0.00 (0)	0.00 (0)	9.95 (57.26)	0.00 (0)	0.45
Total cost of oral antibiotics	0.00 (0)	24.74 (31.57)	0.00 (0)	0.00 (0)	<0.001
Cost of IV antibiotics	700.90 (479.30)	376.93 (331.16)	998.92 (800.40)	667.90 (499.78)	<0.001
Cost per OPAT visit (75 QRs per visit)	0.00 (0)	27.97 (119.72)	2.03 (12.33)	356.25 (88.74)	<0.001
Cost of hospital stay per day	600.00 (241.52)	730.51 (525.97)	2416.22 (3355.14)	675.00 (323.52)	<0.001

IV: Intravenous; OPAT: Outpatient parenteral antimicrobial therapy.

**Table 6.** Additional costs to the hospital (Qatari Riyals).

Treatment option	Mean cost in Qatari Riyals (SD)
Oral antibiotic course	24.9
IV room visit	144.55
Cost of hospital stay per day	2008.81

SD: Standard deviation.
